# Higher theta-beta ratio during screen-based vs. printed paper is related to lower attention in children: An EEG study

**DOI:** 10.1371/journal.pone.0283863

**Published:** 2023-05-18

**Authors:** Michal Zivan, Sasson Vaknin, Nimrod Peleg, Rakefet Ackerman, Tzipi Horowitz-Kraus

**Affiliations:** 1 Educational Neuroimaging Group, Faculty of Biomedical Engineering, Haifa, Israel; 2 Faculty of Education in Science and Technology, Haifa, Israel; 3 Signal and Image Processing Lab, Faculty of electrical engineering, Haifa, Israel; 4 Faculty of Industrial Engineering and Management, Technion, Haifa, Israel; National University of Sciences and Technology, PAKISTAN

## Abstract

Reading is considered a non-intuitive, cognitively demanding ability requiring synchronization between several neural networks supporting visual, language processing and higher-order abilities. With the involvement of technology in our everyday life, reading from a screen has become widely used. Several studies point to challenges in processing written materials from the screen due to changes in attention allocation when reading from a screen compared to reading from a printed paper. The current study examined the differences in brain activation when reading from a screen compared to reading from a printed paper focusing on spectral power related to attention in fifteen 6-8-year-old children. Using an electroencephalogram, children read two different age-appropriate texts, without illustrations, presented randomly on the screen and on a printed paper. Data were analyzed using spectral analyses in brain regions related to language, visual processing, and cognitive control, focusing on theta vs. beta waveforms. Results indicated that while reading from a printed paper was accompanied by higher energy in high-frequency bands (beta, gamma), reading from the screen was manifested by a higher power in the lower frequency bands (alpha, theta). Higher theta compared to the beta ratio, representing challenges in allocating attention to a given task, was found for the screen reading compared to the printed paper reading condition. Also, a significant negative correlation was found between differences in theta/beta ratio for screen vs paper reading and accuracy level in the age-normalized Sky-Search task measuring attention and a positive correlation with performance time. These results provide neurobiological support for the greater cognitive load and reduced focused attention during screen-based compared to print-based reading and suggest a different reliance on attention resources for the two conditions in children.

## Introduction

### Print vs. screen reading

Digital media is everywhere; it is widely used for work and learning purposes as well as in the leisure time of children and adults. With the increasing use of screens (such as computers, e-readers, smartphones, and tablets) for reading over traditional reading from paper, studying the cognitive processes underlying reading comprehension from screens vs. reading from paper has become essential.

Reading ability, decoding written information to spoken sounds in a language, is a relatively new human invention (approximately 5000 years old). As such, to be able to read, the human brain had to "recycle" brain regions and networks developed initially for other cognitive abilities and sensory processes, such as the visual and auditory networks, semantics, and cognitive control [1]. These processes are partially represented in the traditional theoretical model explaining reading comprehension processes, such as the Simple View of Reading (SVR) model [2]. The original version of this model suggested that word decoding and language processing result in reading comprehension [3]. However, recent studies also included sub-components of executive functions in the model as supporting intact reading comprehension [2]. With recent neurobiological evidence of "competing" relations between neural circuits supporting abilities underlying literacy and screen exposure, especially regarding the involvement of executive functions in each process [4–7], a question arises regarding the neurobiological processes underly reading comprehension during printed vs. screen-based text reading.

Meta-analyses examining media effects on reading comprehension examined a complex set of factors that may be related to reading comprehension outcomes (see meta-analyses [8, 9] as well as [10, 11]). These studies consistently report a lower reading comprehension when reading from the screen vs from a printed paper with some exceptions (e.g., narrative texts and hand-held reading devices like Kindle). Notably, age, education level, and experience with digital environments did not moderate screen inferiority factors, indicating that children and adults do not significantly differ in their reaction to it [.e.g., [12]). Screen inferiority was also found in populations who achieve comparable learning outcomes on both media when learning in leisure, limiting the time allowed for reading and testing, as is commonly the case in both work and educational contexts [13]. This suggests the inferior adjustment to task structure (e.g. [13]). Indeed, studies have shown that school-age children show screen inferiority, despite being "digital natives" [14]. A recent meta-analysis suggested that even infants and children aged 1–8 years demonstrate screen inferiority when the texts are comparable on both media [15].

Some explanations for screen inferiority emphasize differences in task design associated with unique opportunities computerized environments provide (e.g., [8]). Such explanation refers, for example, to the "Cognitive load theory" [16, 17], in particular when learning with multimedia [18]. Media-related challenges may be especially pronounced in children, as their information processing, attention, and metacognitive systems are not mature [12, 19–21]. Challenges may also be evident in those with attention disorders [19]. However, screen inferiority was found even with no additional multimedia features, as reviewed in the meta-analyses mentioned above [8, 9], suggesting that a screen-based reading is related to a "sampled" reading (i.e. lower number of fixations during paper vs screen-based reading) [20–22]. This results in a "shallower" processing of the written materials [20–22]. In fact, among adults, less effective processing was found on screens than on paper, even with brief, challenging problem-solving tasks, with 2–3 lines of text, requiring no scrolling or orientation within a page [23]. Interestingly, encouraging deep processing by instructions or task design allowed the same participants to overcome screen inferiority, ruling out screen glare and eyestrain as reasons for this inferiority [23, 24]. These findings suggest that task design and technological differences are not the main sources of screen inferiority, pointing back to the role of executive functions. The persistent screen inferiority in children suggests that cognitive and metacognitive processes (such as attention allocation and effort regulation) may be involved differently during digital-based reading [25]. Understanding the inferior cognitive processing associated with reading comprehension calls for an urgent need to delve into the underpinning mechanisms to focus efforts on overcoming this screen inferiority among children during the critical years in which they acquire learning skills. It will also reinforce the important role of executive functions in the SVR previously suggested.

### Neurobiology of reading from a screen

Most studies examining the neurobiology of reading focused mainly on neurobiological correlates for printed reading [1] and literacy exposure vs. screen reading or exposure time in children [5, 7, 26–28]. Greater functional connections between visual processing regions associated with word recognition (fusiform gyrus) and neural circuits associated with visual and language processing, including speech production regions (Broca), comprehension (Wernicke), and cognitive control regions, were found in association with print reading in 8–12 years old children [5]. On the other hand, more screen-based reading time (including smartphones, tablets, and computers) was associated with decreased functional connections between these regions in these children [5]. In pre-readers, greater exposure to books and reading was associated with increased activation within higher-order visual (e.g., imagery) and cognitive control regions while listening to stories [29]. Moreover, a greater organization of white matter tracts (i.e., a greater fractional anisotropy) connecting these visual, language, and cognitive control regions in 3-5-year-old children was observed [7]. On the other hand, screen viewing in preschoolers was related to the deceased organization of similar white matter tracts [27]. An attempt to compare the two conditions (literacy vs. screen story-viewing) in young children (age 3–5 years) suggested a similar direction as the results above [30]. A greater synchronization between brain networks related to attention and visual processing was found when children were listening to stories and viewed the books’ images (i.e., book pages) vs. when they watched a video of matched illustrations [30]. Overall, these findings support the increased engagement of imagination-related brain regions/networks for the traditional, printed-based literacy exposure vs. screen exposure in children. However, to detect to what extent executive functions processes are involved while comprehending written materials when presented using a screen vs traditional printed paper, the use of tools from the field of neuroscience is warranted.

One of the technical limitations of defining the neural circuits supporting screen-based vs. print-based reading is the challenge of reading from a printed book/paper inside the scanner. Electroencephalogram (or EEG) is another tool that allows overcoming this challenge and comparing brain activity patterns between the two conditions, mainly when focusing on attention allocation processes. Previous EEG studies have shown negative relations between lower attention abilities and higher spectral energy in the lower frequency bands, such as theta and high theta/beta ratios [31–35]. More specifically, lower attention abilities were associated with slow waves activity (especially theta band, between 4–8 Hz) [31–35] together with decreased fast waves activity (beta band between 13–30 Hz and gamma-band between 30–60 Hz) during resting state conditions among 6–18 years old children [32, 36]. Higher connectivity in theta vs. beta bands following six weeks of exposure to recorded videos was observed in preschool children compared to the age-appropriate group exposed to a storyteller, which was related to attention load [28]. This is in line with the higher theta/beta ratio related to mind wandering in healthy adults [37] and to a higher cognitive load [38–42]. Mind wandering, i.e. unrelated thoughts while performing a given task, was negatively related to working memory capacity and reading comprehension abilities in adults [43]. In other words, the greater the working memory capacity is, mind wandering decreases and reading comprehension increases. Mind wandering was also found to be a significant mediator between working memory capacity and reading comprehension, which were driven by attention abilities [44].

On the other hand, focused attention was related to increased spectral power in the higher frequency bands (i.e. beta and gamma) and decreased spectral power in the lower frequency bands (i.e. alpha and theta). More specifically, high spectral power in beta bands was related to better concentration and higher mental activity [45]. Higher levels of visual attention were associated with decreased alpha power and increased beta and gamma powers [46]. The current study’s questions are whether different brain activity patterns during reading from a printed paper vs from a screen are evident in children and whether these patterns (if found) are related to reading comprehension.

The current study aimed to examine the neurobiological signatures for screen-based vs. printed reading using EEG while focusing on executive functions during reading comprehension. To determine the existence of brain and behavior differences between these two conditions, EEG data was collected while children were introduced to a printed text vs a text presented on the screen; both followed reading comprehension questions. We hypothesized that brain waveforms (theta/beta ratio) associated with lower attention allocation and mind wandering would be found in screen vs print-based reading. We also postulated that reading from a printed paper will be accompanied by higher energy in higher frequency bands. In line with that, we also hypothesize that a higher theta/beta ratio for the screen-based vs the printed-based conditions will be found and that this higher theta/beta difference will be associated with a decreased performance in the attention task. Finally, and per previous findings outlined above, we hypothesized higher reading comprehension scores in the printed paper vs the screen reading condition.

## Methods

### Participants

Fifteen 6.41–8.33 years old children (mean age: 7.11 years ± 5.67 months, eight girls) participated in this study (supported by a power analysis including an effect size equivalent to hedges’ g of -0.58 (per [47] supporting our hypothesis of a preference for paper vs screen reading) along with alpha = .05, suggested a minimal number of *N* = 14 participants to reach an 80% power).

All children were typically developing Hebrew-speaking children without known neurological or developmental deficits. The participants were part of a longitudinal study and were recruited through posted advertisements in their former daycares. The Technion’s Institutional ethical committee approved the study, and all methods were performed in accordance with the relevant guidelines and regulations. Each parent signed a written informed consent, and the participants provided verbal assent in line with the ethics committee guidelines. Participants were compensated for their time and travel with a gift at a value of $25.

### Behavioral measures

Each participant performed several behavioral age-normalized tests, including verbal and nonverbal assessments using the Wechsler Preschool and Primary Scale of Intelligence (WPPSI) for children in the first grade and the Wechsler Intelligence Scale for Children (WISC) for children in the second and third grades [48, 49]. Standard scores for the Matrix and Naming subsets were used [48, 49]. Reading ability was assessed using a single-word reading test and a contextual oral reading test from the "Aleph-Taph" battery [50]. Attention abilities were tested using the Sky-search subtest from the Everyday Attention battery for children (TEA-CH) [51]. This behavioral testing session lasted approximately one hour.

### EEG data acquisition

Following the behavioral/neurocognitive data acquisition session, children were invited to participate in the EEG testing in a sound-attenuated room in the lab. After placing the EEG cap, data was recorded while each participant was required to silently read two 100-word expository texts (narratives) followed by five comprehension questions in two conditions: 1) printed text reading and; 2) a screen-based text reading. Comprehension questions included five multiple-choice questions (with four possible answers for each question) related to explicit information noted in the text (for example, several children would be brave and pet the biggest dog in the neighborhood named “Kushi”. One of the questions in this text was: “What was the name of the dog mentioned in the story?”). Both texts were age-appropriate and contained a similar number of words in an equivalent frequency level (texts were taken from [50]). The participant sat in front of a computer screen and read the text from the screen during the screen-reading condition. The computer screen was turned off during the printed text reading, and the child read the text silently from the printed paper. Comprehension questions were answered immediately after the text reading with approximately 4 minutes break before reading the next text. The order of the presentations of the two conditions was randomized.

The EEG recording was performed using 64 electrodes mounted on a custom-made cap (Easy cap, Brain Product, GmbH, Germany) according to the international 10/20 system [52]. The system’s sampling rate was 500 Hz, and an analog bandpass filter with cutoff frequencies of 0.1 Hz and 70 Hz was applied following an A/D conversion with 12 bits. All electrode impedances were maintained under 5 KΩ, due to the temporary repair of the 64-electrode system amplifier. EEG for 4 participants was recorded using an equivalent 16 electrodes of the same manufacturer (Brain Products) using the same caps and electrode sets used in the 64 electrodes system recordings. The 16 electrodes were placed in the following positions: FP1, FP2, F7, F8, Fz, T7, T8, Cz, P7, P3, Pz, P4, P8, O1, Oz, O2.

### Data analyses

#### Behavioral data

Independent *t*-test analyses were conducted for the behavioral tests (verbal and non-verbal tests, reading and attention abilities) to ensure that the participants’ scores were within the normal range.

#### Behavioral measurement: Reading comprehension

A paired *t*-test was conducted to determine differences in reading comprehension in the screen-based vs. print-based conditions.

#### Electrophysiological measurements: Preprocessing

The preprocessing phase of the EEG data was performed using Matlab EEGLAB tool [53]. The preprocessing phase included a manual inspection of the data to eliminate major artifacts. We used a bandpass filter with cutoff frequencies of 0.3 Hz and 45 Hz and a notch filter of 50 Hz. Then, average referencing was performed, computing the average of all electrodes and subtracting it from each. Automatic artifact rejection was performed by detecting abnormally distributed data using the kurtosis measure. Finally, Independent Component Analysis (ICA) was applied to eliminate eye movement components, followed by an inverse—ICA without the removed components.

#### EEG spectral analysis

Spectral data analysis was conducted to examine our hypothesis that reading from printed paper vs screen will be defined with higher energy in higher frequency bands. The spectral analysis was performed by applying the welch method for Power Spectral Density (PSD) calculation and a decimal logarithm operation following [54]. Then, the PSD calculation was summed over each frequency band to calculate the power of each frequency band separately as follows: Delta: .5–3.5 Hz, Theta: 3.5–7.4 Hz, Alpha: 7.5–12.4 Hz, Beta: 12.4–30 Hz, Gamma: 30–45 Hz.

**Fig 1:**
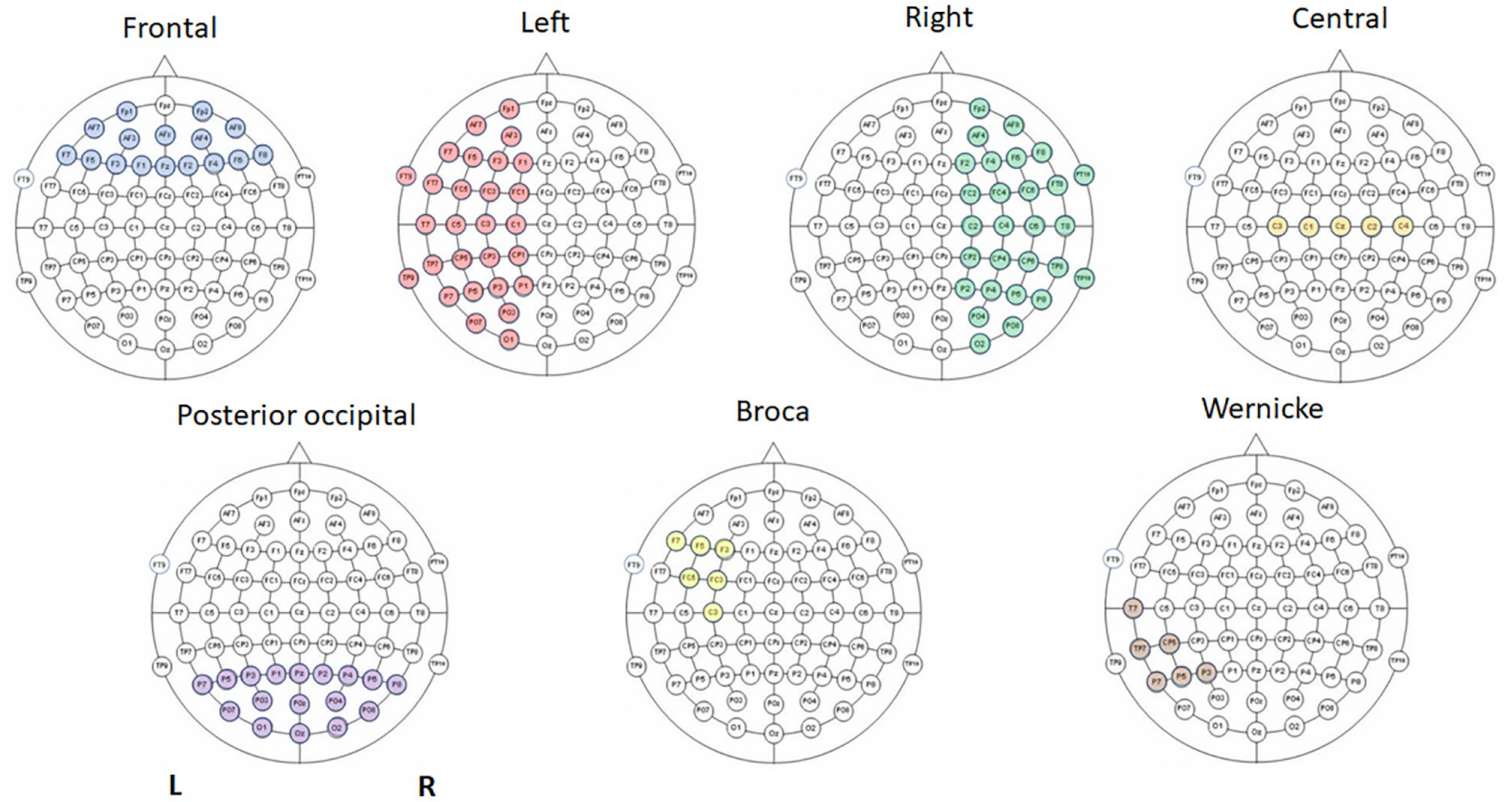
Electrodes position for each ROI: top row (from left to right): frontal, left-hemispheric, right-hemispheric, and central. Bottom row: posterior-occipital, Broca’s area, Wernicke’s area.

The spectral analysis was performed on each electrode separately and a mean was calculated for each of the following regions of interest (ROI) following [55]: frontal (Fp1, Fp2, AF7, AF3, AFz, AF4, AF8, F7, F5, F3, F1, Fz, F2, F4, F6, F8), central (C3, C1, Cz, C2, C4), and posterior-occipital (P7, P5, P3, P1, Pz, P2, P4, P6, P8, PO7, PO3, POz, PO4, PO8, O1, Oz, O2), Broca’s Area (F7, F5, F3, FC5, FC3, C3), Wernicke’s Area (T7, TP7, CP5, P7, P5, P3), total left-hemispheric (Fp1, AF7, AF3, F3, F5, F7, F1, FT9, FT7, FC5, FC3, FC1, C5, C3, C1, T7, TP9, TP7, CP5, CP3, CP1, P1, P3, P5, P7, PO7, PO3, O1), and right-hemispheric (Fp2, AF4, AF8, F2, F4, F6, F8, FT8, FT10, FC4, FC6, FC2, C2, C4, C6, T8, TP8, TP10, CP2, CP4, CP6, P2, P4, P6, P8, PO4, PO8, O2). See Fig 1 for the distribution of the electrodes.

#### Statistical analysis

To test our hypothesis regarding the differences in spectral power density between the two conditions, we performed paired sample *t*-tests between the two conditions of the spectral power calculated for each frequency band and each ROI.

#### Correlations between EEG and behavioral measures

To determine the relations between cognitive and electrophysiological measures associated with visual attention and attention load, Spearman correlations between attention abilities (using the accuracy and time measures from the Sky-Search subtest from the Tea-Ch battery) and theta/beta ratio during screen vs paper-based reading were conducted.

## Results

### Behavioral test

The behavioral assessment for verbal and nonverbal IQ as well as reading skills indicated that all participants were within the normal range. See Table 1.

**Table 1:** Behavioral and cognitive measures (mean and standard deviation).

Measure	*M*	*SD*	Normal range	Test reliability (α-Cronbach)
General IQ (WPPSI/WISC, Matrix, standard score)	11.6	2.94	7–13	0.95
Verbal ability (WPPSI/WISC, Naming, standard score)	9.86	2.97	7–13	0.86
Reading words ("Aleph-Taph", Single-word reading, number of words per minute)	26.69	14.17	12–51	0.9
Text reading, speed ("Aleph-Taph", reading per minute, standard score)	-0.51	1.02	-1.55–1.19	0.79
Text reading, accuracy ("Aleph-Taph", reading mistakes, standard score)	-0.22	0.99	-0.64–0.61	0.88
Attention, accuracy (TEA-CH, Sky-search, percentile)	47	27	25–75	0.73

M = Mean, SD = Standard deviation

Reading comprehension results

No significant differences in comprehension scores between the two conditions were found (print reading: *M* = 3.75, *SD* = 1.21, screen reading: *M* = 4.08, *SD* = 1.16, *t* = 1.076, *p* = .305).

### EEG measures results

#### Spectral analysis

**Fig 2:**
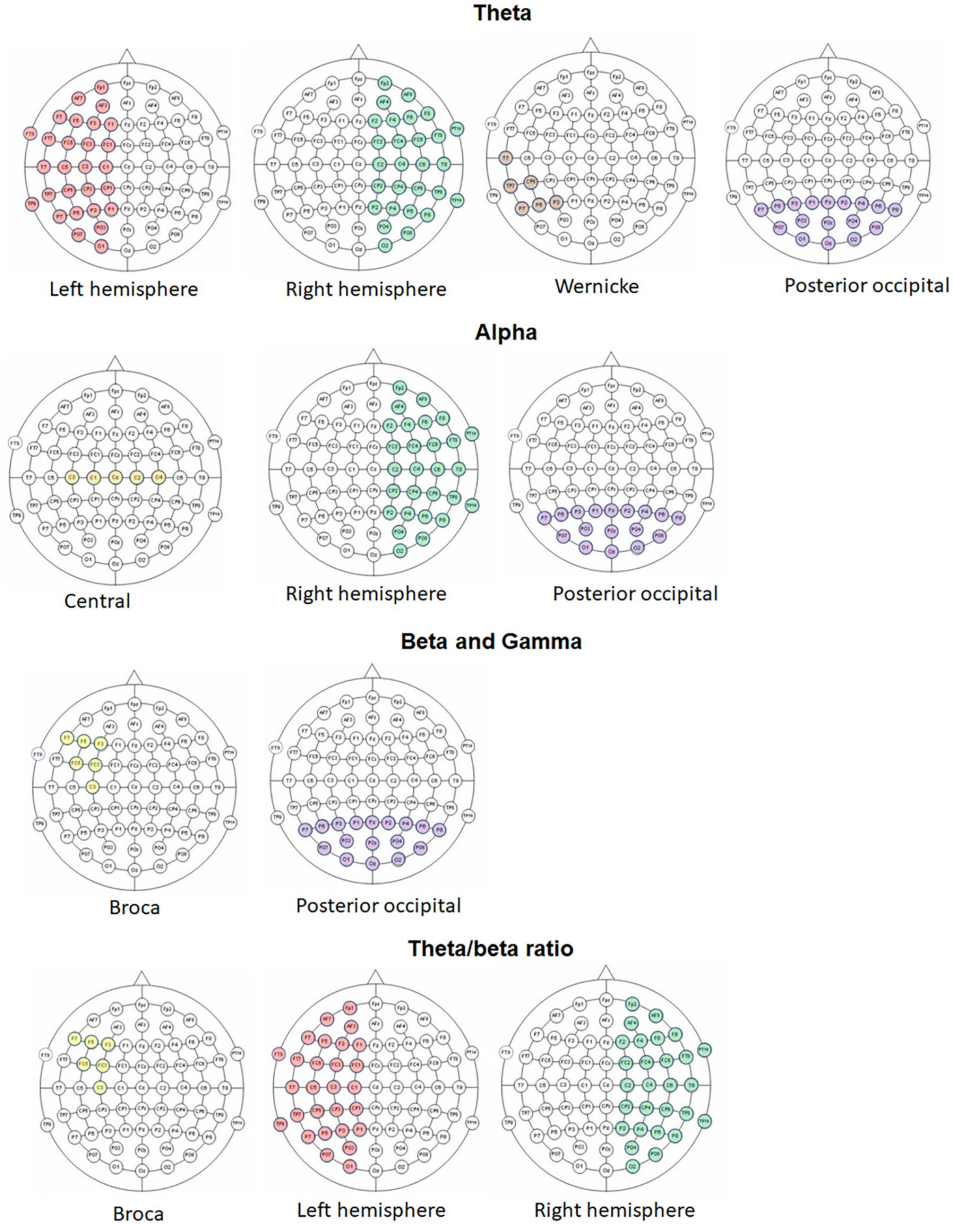
A visualization for the location of the electrodes showing significant differences between the reading conditions per frequency band.

A paired sample *t*-test analysis was conducted between the mean spectral power in each ROI for each of the frequency bands for the printed vs screen-based reading (See Table 2). Results show that higher spectral power in the lower frequency bands (theta and alpha) were found when reading from screen vs. reading from paper in the following locations: Theta in the left (*p* = .008) and right (*p* = .001) electrodes and posterior occipital (*p* = .00) electrodes and those covering Wernicke (*p* = .015); in the alpha band: central (*p* = .041), posterior occipital (*p* = .012) and right-lateralized locations (*p* = .037). Additionally, higher spectral power in the higher frequency bands (beta and gamma) were observed when reading from paper vs. reading from the screen (beta bands: posterior (*p* = .014) and Broca locations (*p* = .04); gamma band: posterior occipital (*p* = .001), Broca (*p* = .001) and left-lateralized locations (*p* = .011)). Theta vs. beta ratio was significantly higher for the screen vs. print-reading in the left (*p* = .008), right (*p* = .006), posterior (*p* = .00), and Broca locations (*p* = .001). See Table 2 and Fig 2.

**Table 2:** Mean Decimal logarithm of Power Spectral Density (PSD) for paper vs screen-based reading in the different frequency bands (Delta, Theta, Alpha, Beta, and Gamma: A-E), Theta/beta ratio (F), and the different brain regions (frontal, central, left hemisphere, right hemisphere, posterior-occipital, Wernicke and Broca) and Delta (0.5–3.5 [in Hz]).

A.							
	Frontal electrodes *M(SD)*	Central electrodes *M(SD)*	Left Hemisphere electrodes *M(SD)*	Right Hemisphere electrodes *M(SD)*	Posterior Occipital electrodes *M(SD)*	Wernicke electrodes *M(SD)*	Broca electrodes *M(SD)*
Paper	10.05(0.51)	9.21(0.80)	9.73(0.64)	9.57(0.64)	9.36(0.85)	9.92(0.82)	9.70(0.80)
Screen	10.02(0.53)	9.28(0.74)	9.65(0.65)	9.53(0.75)	9.34(0.83)	9.84(0.83)	9.52(0.78)
*T*(*p*)	-0.26(.8)	0.7(.5)	-0.89(.39)	-0.43(.67)	-0.44(.66)	-1.12(.28)	-1.19(.25)
B. Theta (3.5–7.4 [Hz])							
Paper	10.67(0.79)	9.95(1.24)	10.30(0.99)	10.17(0.96)	10.01(1.26)	10.38(1.15)	10.31(1.17)
Screen	10.80(0.73)	10.22(1.03)	10.48(0.95)	10.42(0.99)	10.38(1.23)	10.52(1.14)	10.37(1.06)
*T*(*p*)	1.28(.24)	1.55(.14)	**3.1(.008)**	**4.35(.001)**	**5.82 (= .00)**	**2.76(.015)**	0.49(.63)
C. Alpha (7.5–12.4 [Hz])							
Paper	11.12(1.23)	10.59(1.64)	10.91(1.55)	10.74(1.43)	10.67(1.83)	11.11(1.95)	10.98(1.76)
Screen	11.28(1.17)	11.06(1.45)	11.09(1.46)	11.00(1.53)	11.03(1.81)	11.33(1.91)	11.02(1.63)
*T*(*p*)	1.1(.29)	**2.25(.04)**	1.42(.18)	**2.31(.037)**	**2.88(.01)**	1.73(.11)	0.21(.84)
D. Beta (12.4–30 [Hz])							
Paper	30.19(5.23)	24.74(6.29)	28.88(6.47)	28.36(5.64)	26.84(6.66)	29.18(10.64)	28.77(6.65)
Screen	30.34(5.00)	24.96(5.94)	27.74(6.09)	27.77(5.50)	25.66(6.59)	30.73(9.64)	26.82(6.14)
*T*(*p*)	0.38(.71)	0.43(.68)	-1.88(.08)	-1.7(.11)	**-2.8(.01)**	1.28(.22)	**-2.26(.04)**
E. Gamma (30–45 [Hz])							
Paper	19.92(5.89)	14.06(6.45)	19.24(6.93)	28.36(5.64)	17.83(6.80)	20.47(14.49)	19.09(6.43)
Screen	19.40(5.67)	12.67(6.20)	16.72(6.09)	27.77(5.50)	14.50(6.32)	19.95(10.83)	15.87(5.66)
T(p)	-0.88(.4)	-2(.065)	**-2.92(.01)**	-1.69(.11)	**-4.14(.001)**	-0.63(.54)	**-3.45(.004)**
F. Theta/ beta							
Paper	0.36(0.04)	0.42(0.07)	0.37(0.06)	0.37(0.05)	0.39(0.06)	0.40(0.16)	0.37(0.06)
Screen	0.36(0.04)	0.42(0.07)	0.39(0.06)	0.38(0.05)	0.42(0.07)	0.36(0.07)	0.40(0.06)
*T*(*p*)	0.43(.67)	1.24(.23)	**3.07(.008)**	**3.25(.006)**	**4.56 (= .00)**	-0.98(.34)	**4(.001)**

Mean (*M*), standard deviations (*SD*), *t* and significance values (*p*) for the different frequency bands in each brain location.

#### Correlations between theta/beta electrophysiological measures for screen vs paper reading and behavioral attention abilities

Spearman correlation between the accuracy and time levels from the sky-search task and the difference between theta/beta ratio during screen vs paper-based reading revealed 1)a significant negative correlation for the difference between theta/beta ratio in the two conditions in Wernicke and accuracy percentage for the attention task (r = -0.556, p = .014) and; 2)a positive correlation between theta/beta ratio and time per correct target (r = .643, p = .005). Results suggest that a higher theta/beta ratio for the screen compared to the printed paper reading condition is related to a decreased performance in this attention task and a longer time to reach an accurate response in this task.

## Discussion

Increasing/extensive research has found differences in cognitive and neural processing of narratives in print vs screen format [9, 10]. The goal of the current study was to determine the differences in the involvement of executive functions and attention allocation/load during printed paper (previously reported, per the updated SVR [3]) vs screen-based reading among children. In line with our hypotheses, printed-paper reading was accompanied by significantly greater energy in the higher frequency bands (beta, gamma), while reading from the screen was related to lower frequency bands (theta, alpha). Additionally, as expected, a greater theta/beta ratio was observed during the screen-based vs the printed-paper condition, which was also negatively correlated with visual attention abilities. These results, which were previously related to cognitive load [38–42], attention difficulties, metacognitive processes, mind wandering, and exposure to screens [see [12, 23, 28, 32, 33, 37, 56]], were found in most tested topographical regions. More specifically, McVay and Kane [44] demonstrated how mind wandering is a mediator between working memory capacity and reading comprehension, emphasizing the connection between attention control over thoughts and reading comprehension [44]. Media differences in brain activity included Broca, left and right localizations, and posterior and were most pronounced in the posterior-occipital ROI, which contains the visual processing areas. Here, the relations between EEG findings for the two reading conditions and cognitive load, attention abilities and mind wandering will be discussed.

### Screen inferiority in children’s focus

Previous literature suggested that higher concentration and visual attention are associated with higher spectral power in the higher frequency bands (i.e., beta and gamma) [45, 46]. In contrast, attention difficulties and mind wandering were associated with higher spectral power in the lower frequency bands (i.e., alpha and theta) [31–35, 37], as well as a higher theta beta ratio [32, 33, 56]. In addition, exposure to screens was shown to reduce attention span and increase learning difficulties and behavioral problems among children [57–59]. This was supported by neuro-correlate evidence of increased connectivity in theta vs. beta bands after increased exposure to screens [28]. Integrating this literature with the current study results suggests that when the text is presented to children via screens, the children present brain activity patterns that indicate more "daydreaming", and less focused attention. In contrast, when the same children read a text from a paper, they present a more concentrated brain activity pattern. Interestingly, this higher theta/beta ratio for screen vs paper reading in electrodes associated Wernicke area (related to comprehension) was associated with longer performance time and lower accuracy rate while performing the attention task. To the best of our knowledge, these results constitute the first neuro-correlate support for previous behavioral studies [8, 14, 15, 20, 60, 61]. These results point at the advantages of reading from paper in higher-order comprehension, speed, and lower fatigue than when reading on screens.

Children’s attention is overloaded when exposed to screens.

The "cognitive-load theory" suggests that the processing of sensory information has a limited capacity, and hence information overload may interfere with processing the written information [15]. As noted earlier [15], exposure to screens attracts children’s attention with a trade-off of information processing quality. The attraction of attention can either be an actual interference such as banners, sounds, touch-based activities, or the expectation that the content will be interactive [15]. This expectation may increase the demand for attention resources allocated towards comprehension (even with the lack of interaction in the current study). However, our study did not find media effects on comprehension levels, as hypothesized. This might be due to the relatively "sterile" nature of the text presented on the screen in the current study rather than a web-based or tablet-based reading involving sensory-motor stimulation. It may also stem from the self-regulated learning allowed in the present study, without any time frame, that allowed children to compensate for the higher demand in computerized learning [8]. Indeed, a higher cognitive load was observed in the screen-based condition as manifested by the higher spectra power in the Theta frequency band relative to paper-based learning. Therefore, our EEG results provide neurobiological support for the increased attention demands involved in screen reading vs paper-based previously demonstrated by behavioral studies among children [12, 15, 62] and adults with attention deficits [19]. The fact that no behavioral differences were found for reading comprehension between the two conditions is of interest as the EEG data provided information on mechanisms that underlie the reading comprehension process, which may play even greater role in special populations (those with reading or attention challenges) and in longer, more complex task reading. Interestingly enough, and although against our hypothesis, our results of the lack of reading comprehension differences between the two conditions are supported by the meta-analyses by Delgado et al. (2018) [8] and Clinton (2020) [63], who found no screen inferiority when reading narrative texts. This lack of difference in reading comprehension while reading narratives is explained by [64, 65], who suggest that expository texts depend on background knowledge for inferences, whereas narratives often do not. Hence, it may be that the text-reading platform doesn’t dramatically affect reading comprehension when focusing on narratives. It would be of interest if differences in comprehension would also be found for expository text reading among children.

### Less involvement of imagination during screen-based reading?

Reading comprehension involves higher-order cognitive abilities and attention allocation [Per Kintch model for the connectionist model for reading comprehension [66, 67], and the SVR model [2]]. Uniquely and similarly to narrative comprehension, reading comprehension also involves visualization, which was also manifested with the activation of visual regions in 5-year-old children listening to stories in relation to their later reading comprehension scores [68]. Results from previous studies showed that the brain regions engaged in imagining the stories the participants heard at the age of 5 were related to greater reading comprehension scores at the age of 11 [69]. Visualization is a mechanism children use when listening to stories, even in a more comprehensive manner than when children watch an animated video [26] and is utilized as children listen to more stories in their homes [70] (for review of additional studies involving visualization during reading comprehension, see [71, 72]). However, to date, no studies have examined the level of visualization while reading from a static computer screen vs a traditional print-based reading. Our previous results demonstrated decreased engagement of attention and visual processing networks associated with imagination while watching a video versus listening to a story [30]. In relation to the current study, it may be interesting examining the reliance on visualization strategies while comprehending written materials from the screen vs from a paper. Our results of theta/beta ratio on posterior occipital brain regions for screen vs paper reading and the negative correlations with visual attention abilities may point to this direction but still, need further support.

Another interesting difference between screen and paper reading is the reduced level of sensory-motor stimulation while reading from the screen, at least when reading on a computer screen (which was the condition used in the current study) (see [6]). Sensory-motor stimulation is essential for reading and is related to embodiment, i.e. our body’s interaction with the environment in two aspects: Spatio-temporal (body movement and location during reading) and imagery (the role of our body while imagining the narrative during reading explained earlier). It was suggested that the lack of materiality during screen reading (as opposed to paper/book reading) affects the above aspects of embodiment during reading, which in turn affects the way we interact with the text [7]. This change in embodiment during reading may be related to reading comprehension level [8]. The relatively short narratives used in this study may not have an effect on reading comprehension levels but may result in a greater cognitive overload. It would be interesting to examine if differences may open up between the two conditions when the text complexity and length increase or for a different genre.

### Studies limitations and future directions

The current study has some limitations that should be taken into account: First, reading comprehension can be assessed at different levels. This study used questions determining basic text understanding, which showed no differences between the media. There may be differences in higher-order comprehension levels, such as conclusion drawing and inferences, that were not tested here. Second, screen-based presentation of information typically includes more interactive media, web-based, or even Kindle, which involves the sensory-motor modality. With previous studies showing better comprehension levels during reading from an interactive media screen vs still screen [73], additional research should be done to assess these conditions’ neurobiological footprint. Third, in the current study, we related to previous studies associating reading from a screen with mind wandering [21], while these studies measured the level of mind wandering by interrupting the participants during reading and asking them about their thoughts during a given moment. As the participants in the current study were young children, interrupting them during the reading process might harm their comprehension. However, to relate the changes in EEG found in the current study to mind wandering- a direct measurement of this process should take place. Also, several studies suggested on-screen inferiority due to different visual scanning patterns] more and short fixations and lower fatigue levels when reading from a paper [74][. Future studies combining EEG and eye-tracking should be conducted to associate the reading pattern with the EEG waveforms. An fMRI study should be conducted to understand better how the current research results are represented in the spatial space, i.e., visual processing, cognitive control networks or language areas and the level of reliance on attention/cognitive control networks rather than correlating the waveforms with attention abilities, as was done here. This study enrolled fifteen children, which may impact the lack of reading comprehension differences in the two conditions (despite the support for the lack of expected differences in the literature [8, 63]. Hence, a larger scale study should be conducted to be able to generalize these results on larger groups of children in different age groups. It is also important to emphasize that the current study used short texts rather than complex, long ones. Additional studies examining the effect of reading texts of different lengths and genres are needed to better understand the effect of reading in these two modules on cognitive load and comprehension.

## Conclusions

EEG and behavioral findings in this study suggest differences favoring paper vs screen-based format in children. These results support the AAP guidelines regarding the need to limit screen exposure to young children, with a possible need to consider employing cognitive control and self-regulation activities when reading from screens, with particular consideration given to beginning readers. Our results also reinforce the critical role of executive functions and attention allocation during reading comprehension, as stated in the updated SVR model [2].

## Supporting information

S1 Data (XLS)
